# Ultrasound-Assisted PLC/PLA_2_ Enzymatic Degumming of Rapeseed Oil: Process Optimization, Lipidomic Analysis, and Storage Stability

**DOI:** 10.3390/foods15203751

**Published:** 2026-10-21

**Authors:** Kang Lin, Conghui Lang, Yanpei Huang, Yukun Han, Weijun Chen, Wenxue Chen, Jianfei Pei, Ying Lv, Rongrong He, Haiming Chen, Ming Zhang

**Affiliations:** 1 Hainan University-HSF/LWL Collaborative Innovation Laboratory, College of Food Sciences & Engineering, Hainan University, 58 People Road, Haikou 570228, China; lk120712@163.com (K.L.); 13103951695@163.com (C.L.); hyp173859463492022@163.com (Y.H.); chenwj@hainan.edu.cn (W.C.); chwx@hainanu.edu.cn (W.C.); peijianfei@hainanu.edu.cn (J.P.); lyuying@hainanu.edu.cn (Y.L.); rongronghe@hainanu.edu.cn (R.H.); hmchen168@126.com (H.C.); 2 Hainan AUSCA International Grains and Oils Co., Ltd., Yangpu Free Trade Port, Yangpu Economic Development Zone, Danzhou 578001, China; asklyxz@126.com

**Keywords:** rapeseed oil, ultrasound, enzymatic degumming, storage stability

## Abstract

Degumming is a critical step in rapeseed oil (RO) refining, directly affecting subsequent processing efficiency and oil quality. This study optimized an ultrasound-assisted composite enzyme (phospholipase C/phospholipase A_2_, PLC/PLA_2_) degumming process using single-factor experiments and response surface methodology. Under optimal conditions, the residual phosphorus content was reduced to 4.55 ± 0.23 mg/kg. LC–MS/MS analysis revealed that the major phospholipids in crude RO were phosphatidylcholine (PC), phosphatidylglycerol (PG), and phosphatidylethanolamine (PE), with the ultrasound-assisted enzymatic degumming (UEDAC) process achieving a phospholipid removal rate of 98.19%. Lipidomics analysis further showed that UEDAC primarily influenced the composition of glycerophospholipids (GPs) and glycerides (GLs). Furthermore, this process effectively reduced residual calcium and magnesium contents, while unsaturated fatty acids remained the predominant fatty acid fraction after degumming. During accelerated storage, UEDAC-treated oils exhibited improved oxidation stability, enhanced antioxidant capacity, and better retention of nutritional components. These results indicate that UEDAC is an effective strategy for improving the degumming efficiency and quality of RO.

## 1. Introduction

Rapeseed oil (RO) is an important edible vegetable oil obtained from rapeseed via mechanical pressing or solvent extraction and is one of the major vegetable oils worldwide. In China, RO is an important component of the edible oil supply, ranking third in production after soybean oil and palm oil. Its wide application in household cooking and the food processing industry highlights its economic and nutritional value [1]. RO is not only an economically important edible oil but is also valued for its favorable fatty acid composition. Compared with many other dietary fats, RO is relatively low in saturated fatty acids and is dominated by unsaturated fatty acids, which account for nearly 90% of the total fatty acid fraction. Among these, oleic acid (C18:1), linoleic acid (C18:2 n − 6), and α-linolenic acid (C18:3 n − 3) are the major components. The high proportion of unsaturated fatty acids contributes markedly to the favorable nutritional profile of rapeseed oil. In particular, linoleic acid and α-linolenic acid are essential fatty acids that are not synthesized by the human body in sufficient amounts and therefore need to be supplied through the diet. Their presence further enhances the dietary significance of RO [2]. In addition, RO contains various trace bioactive components, such as tocopherols, phytosterols, and carotenoids. These components may contribute to maintaining metabolic homeostasis through antioxidant activity, regulation of inflammatory responses, and alleviation of oxidative stress. Existing research indicates that RO may help reduce the risk of diabetes, hypertension, and other chronic diseases [3].

Before RO is marketed, it typically undergoes a series of refining processes to remove phospholipids, free fatty acids, pigments, off-flavor compounds, and other impurities that affect oil quality and stability. As the first step in refining, degumming is mainly responsible for removing phospholipids and related gum substances. Its efficiency not only determines the performance of subsequent deacidification, decolorization, and deodorization processes but also influences the color, flavor, and oxidative stability of the final oil [4]. Although water degumming and acid degumming are widely applied in RO refining, their phosphorus removal efficiency is often insufficient to continuously reduce phosphorus levels below 10 mg/kg [5]. Moreover, these conventional processes may generate phosphorus-containing wastewater or acidic effluents, increasing the burden of wastewater treatment and leading to the loss of certain nutrients and bioactive components [2]. Compared with traditional approaches, enzymatic degumming is considered a greener alternative. It employs phospholipases to hydrolyze phospholipids into more easily separable products, thereby improving phosphorus removal efficiency under milder conditions while reducing chemical consumption and wastewater generation [6]. In recent years, combined phospholipase systems have received increasing attention [2]. These approaches enhance the synergistic hydrolysis and transformation of phospholipid species by combining phospholipases with different action sites and substrate spectra, thereby enhancing degumming efficiency. However, most existing studies have focused primarily on reducing phosphorus content, while systematic evaluations of the overall quality and storage stability of degummed oil remain limited. Therefore, further research is needed to clarify the quality characteristics of degumming oil and its changes during storage.

Ultrasonic waves have been recognized as an efficient auxiliary technology in oil processing and have attracted increasing attention in vegetable oil refining in recent years [7]. The introduction of ultrasound into enzymatic degumming may generate cavitation effects at the oil–water interface, thereby promoting the dispersion of phospholipases at the interface. This facilitates the production of oil with a lower residual phosphorus content. Kalenchak et al. reported that in RO systems, ultrasound-assisted treatment enhances enzymatic degumming efficiency while reducing water usage, reaction time, and enzyme dosage [8]. Similarly, More et al. investigated ultrasound-assisted enzymatic degumming of crude soybean oil and reported that, under optimal conditions, degumming efficiency could reach 98.4% [9]. However, some studies have shown that excessive ultrasonic intensity may induce local overheating and promote the formation of free radicals, thereby accelerating lipid oxidation [10,11]. Therefore, changes in oxidative stability should be carefully monitored during ultrasound-assisted degumming. At present, systematic optimization studies on ultrasound-assisted enzymatic degumming of RO remain limited, and its specific effects on oil quality attributes and storage stability are not yet fully understood. Accordingly, further systematic research is needed to clarify the relationships among ultrasonic conditions, degumming efficiency, oil quality, and storage stability.

This study aims to optimize ultrasound-assisted degumming of rapeseed oil (RO) using a PLC/PLA_2_ enzyme combination by examining the effects of water content, pH, reaction temperature and time, ultrasonic power, calcium chloride dosage, and enzyme ratio. It further aims to evaluate the effects of the optimized degumming process on the metal ion content and fatty acid composition of RO and to use lipidomics to characterize changes in lipid molecular profiles associated with the degumming process. The study also aims to evaluate changes in oil quality, antioxidant capacity, and the retention of bioactive components in degummed RO during accelerated storage. Overall, this study seeks to establish an efficient ultrasound-assisted enzymatic degumming process for RO and to clarify the effects of PLC/PLA_2_ composite enzymatic degumming on oil quality characteristics and quality preservation during accelerated storage.

## 2. Materials and Methods

### 2.1. Materials

RO, containing an initial phosphorus level of 251.86 mg/kg, was provided by Hainan Ausca International Grain & Oil Co., Ltd. (Hainan, China). Phospholipase A_2_ (PLA_2_; 10,000 U/mL) and phospholipase C (PLC; 200 U/g) were obtained from Shanghai Huashang Xiangyang Biotechnology Co., Ltd. (Shanghai, China). Methanol, acetonitrile, methyl tert-butyl ether (MTBE), ammonium formate, and isopropanol were supplied by CNW Technologies GmbH (Düsseldorf, Germany). Unless otherwise specified, all remaining chemicals were of analytical grade and sourced from Sinopharm Chemical Reagent Co., Ltd. (Shanghai, China).

### 2.2. Crude Oil Degumming Methods

The degumming procedure was adapted from the method described by Liu et al. [12]. with minor modifications. A 100 g portion of crude RO was preheated to 65 °C and combined with 1 mL of 45% (*w*/*w*) citric acid solution. After shear mixing at 2000 rpm for 1 min, the oil was cooled to the required reaction temperature, and its pH was brought to the preset value using 1 mol/L NaOH solution. According to the corresponding experimental treatment, prewarmed deionized water, the enzyme preparation providing a total activity of 150 U per 100 g oil, and CaCl_2_ solution were sequentially incorporated into the system. For different PLC (a) to PLA_2_ (b) ratios, the activities of PLC and PLA_2_ were calculated as 150a/(a + b) and 150b/(a + b) U, respectively. The mixture was then homogenized at 8000 rpm for 1 min and subjected to enzymatic degumming under continuous ultrasonication in an ultrasonic bath (KQ-600DE, Kunshan Ultrasonic Instruments Co., Ltd., Kunshan, China; frequency, 40 kHz; rated ultrasonic power, 600 W), with the ultrasonic output (50–90% of the rated power), temperature (40–60 °C), and treatment time (30–90 min) set according to the corresponding experimental conditions. At the end of the reaction, the temperature was increased to 85 °C and maintained for 15 min to deactivate the enzymes. Phase separation was subsequently achieved by centrifugation at 7000 rpm for 10 min, and the oil layer obtained after centrifugation was collected as the degummed RO.

### 2.3. Optimization of the Degumming Process Using Single-Factor and RSM Methods

To optimize the process parameters for ultrasound-assisted enzymatic degumming of RO, a single-factor experimental design was first employed. Moisture content was selected as the initial variable and set at 1%, 2%, 3%, 4%, and 5% (*w*/*w*, based on oil weight), while other conditions were kept constant at pH 6, 50 °C, continuous ultrasonication at 40 kHz with the ultrasonic output set at 70% of the rated power, a reaction time of 45 min, a CaCl_2_ addition level of 0.07% (*w*/*w*, based on oil weight), and an enzyme ratio (PLC:PLA_2_) of 1:1. The PLC and PLA_2_ enzyme mixture used in this study had a combined initial activity of 150 U before being introduced into the degumming system. The reported enzyme activity represents the initial activity prior to the ultrasonic-assisted degumming treatment. Subsequently, the effects of pH (4, 5, 6, 7, 8), temperature (40, 45, 50, 55, 60 °C), ultrasonic output (50%, 60%, 70%, 80%, and 90% of the rated power), reaction time (30, 45, 60, 75, 90 min), CaCl_2_ addition level (0.03%, 0.04%, 0.05%, 0.06%, and 0.07%), and enzyme ratio (PLC: PLA_2_ = 3:1, 2:1, 1:1, 1:2, and 1:3) on degumming performance were systematically evaluated. In each experiment, only one factor was varied while all other conditions were held constant.

Response surface methodology (RSM) was employed after completion of the single-factor trials. Moisture content, pH, and temperature are key reaction-environment parameters affecting phospholipase-mediated degumming and were therefore selected for multivariate optimization. Based on the single-factor results, the levels of the three selected factors were set as follows: moisture content, 3%, 4%, and 5%; pH, 5, 6, and 7; and reaction temperature, 40, 45, and 50 °C. All remaining process variables were maintained at the optimum conditions identified in the preliminary single-factor tests. Design-Expert 12.0 software (Stat-Ease Inc., Minneapolis, MN, USA) was used to assess the individual and interactive effects of these factors on the response variable, residual phosphorus content (Y). The experimental design comprised 17 runs, and each treatment was conducted in triplicate. Mean values obtained from the three replicates were used for further statistical analysis.

### 2.4. Phosphorus Content Analysis

Phosphorus content was determined according to the method of Pan et al. [13], with minor modifications. An oil sample (10.0 g) was mixed with 0.5 g of zinc oxide in a porcelain crucible, pre-carbonized on an electric furnace, and subsequently incinerated at 550 °C for 2 h. After cooling to room temperature, the resulting ash was dissolved in 10 mL of hydrochloric acid–water solution (1:1, *v*/*v*), gently boiled for 5 min, filtered, and brought to a final volume of 100 mL with deionized water. A 10 mL portion of the filtrate was neutralized using 50% potassium hydroxide solution, after which hydrochloric acid–water solution was added until the precipitate was completely redissolved. Subsequently, 8 mL of 0.015% hydrazine sulfate solution and 2 mL of sodium molybdate–sulfuric acid color-developing reagent were introduced. The reaction mixture was maintained in a boiling water bath for 10 min, cooled to room temperature, and diluted to an appropriate volume. The absorbance was then recorded at 650 nm.

### 2.5. Lipidomics Analysis

#### 2.5.1. Sample Pretreatment

Lipids were extracted according to the method described by Matyash et al. [14], with minor modifications. After the RO sample had been thoroughly homogenized, 0.020 ± 0.001 g was transferred into a 2 mL centrifuge tube preloaded with one steel bead. Lipids were extracted by adding 1.0 mL of methyl tert-butyl ether/methanol (3:1, *v*/*v*), followed by mechanical agitation at 2500 rpm for 15 min at room temperature. Phase separation was initiated by introducing 300 μL of ultrapure water and vortexing the mixture for 1 min. The sample was subsequently held at 4 °C for 10 min and centrifuged at 12,000 rpm for 10 min. From the separated phases, 200 μL of the upper organic fraction was withdrawn and dried under vacuum at 20 °C. The residue was then dissolved in 200 μL of acetonitrile/isopropanol (1:1, *v*/*v*), mixed by vortexing for 3 min, and centrifuged again at 12,000 rpm for 3 min at 4 °C. A final volume of 120 μL of the clarified supernatant was placed in an LC–MS vial and used for LC–MS/MS measurement.

#### 2.5.2. LC–MS/MS Conditions

Lipid analysis was performed by LC–MS/MS based on previously reported methods [15,16]. An ExionLC™ AD UPLC system coupled to a QTRAP^®^ 6500+ tandem mass spectrometer (SCIEX, Framingham, MA, USA) was used. Lipid components were separated using a Thermo Accucore™ C30 chromatographic column (2.6 μm, 2.1 mm × 100 mm; Thermo Fisher Scientific, Waltham, MA, USA). Eluent A contained acetonitrile and water at a ratio of 60:40 (*v*/*v*), with 0.1% formic acid and 10 mmol/L ammonium formate added as modifiers. Eluent B was prepared from acetonitrile and isopropanol (10:90, *v*/*v*) and supplemented with identical concentrations of formic acid and ammonium formate. Gradient elution proceeded according to the following schedule: 80% A and 20% B at 0 min; 70% A and 30% B at 2 min; 40% A and 60% B at 4 min; 15% A and 85% B at 9 min; 10% A and 90% B at 14 min; 5% A and 95% B from 15.5 to 17.3 min; and restoration to 80% A and 20% B from 17.5 to 20 min. Throughout the analysis, the eluent was delivered at 0.35 mL/min, the column was thermostated at 45 °C, and 2 μL of sample was injected.

Electrospray ionization was used for mass spectrometric detection, with data acquired in both positive and negative ionization modes. The ion-source temperature was maintained at 500 °C, while the spray voltages were adjusted to 5500 V in positive mode and −4500 V in negative mode. Gas pressures were set to 45 psi for ion source gas 1, 55 psi for ion source gas 2, and 35 psi for the curtain gas. Lipid analytes were detected in multiple reaction monitoring (MRM) mode, and each transition was analyzed using individually optimized declustering potential and collision energy settings. Lipid species were semi-quantified using an internal-standard-based approach.

#### 2.5.3. Data Processing

Raw MS data files were converted to mzXML format using the msConvert program from ProteoWizard (version 3.0). The converted data were subsequently processed using XCMS (version 3.2), with the CentWave algorithm employed for peak detection, extraction, retention-time correction, alignment, and integration. The minfrac and cutoff parameters were set to 0.5 and 0.3, respectively. Lipid annotation was performed by matching the acquired MS/MS spectra against the LipidBlast spectral library using an R-based workflow built on XCMS.

### 2.6. Metal Ion Analysis

Metal elements were determined according to the method of Jiang, Chang, Wang, Jin and Wang [17]. Approximately 0.8 g of each sample was accurately weighed and digested with 5 mL of aqua regia (HCl: HNO_3_ = 3:1, *v*/*v*) using a CEM MARS 6 microwave digestion system (CEM Corporation, Matthews, NC, USA), the temperature was increased to 180 °C and held for 30 min. After digestion, the digest was brought to a final volume of 10 mL with ultrapure water. Calcium and magnesium concentrations in the samples were quantified using an iCAP TQ inductively coupled plasma mass spectrometer (ICP–MS; Thermo Fisher Scientific, Waltham, MA, USA). The operating conditions were as follows: radio-frequency power, 1550 W; plasma gas flow rate, 14 L/min; auxiliary gas flow rate, 0.7763 L/min; nebulizer gas flow rate, 1.0749 L/min; spray chamber temperature, 2.5 °C; peristaltic pump speed, 40 rpm; dwell time, 0.1 s; and three replicate acquisitions. Data acquisition was conducted under kinetic energy discrimination (KED) mode.

### 2.7. Fatty Acid Composition Analysis

Fatty acid methylation was performed according to the method of Cui et al. [18]. For the preparation of fatty acid methyl esters, 2 mg of the sample was dissolved in 1.5 mL of hexane, after which 100 μL of methanolic sodium methoxide and 40 μL of methyl acetate were introduced. The mixture was maintained at room temperature for 20 min to allow methylation to proceed and was then chilled at −20 °C for 10 min. Next, 60 μL of oxalic acid solution was added, and the sample was centrifuged at 4200 rpm for 10 min. The clear supernatant was passed through an anhydrous sodium sulfate column to remove traces of moisture. The collected fraction was concentrated to dryness under a gentle nitrogen stream, yielding the fatty acid methyl ester derivatives. Prior to instrumental determination, the dried derivatives were dissolved in 1 mL of hexane and transferred into an autosampler vial.

Fatty acid composition was analyzed on an Agilent 7890A gas chromatographic system (Agilent Technologies Inc., Santa Clara, CA, USA) equipped with a flame ionization detector and a CP-Sil 88 capillary column (100 m × 0.25 mm, 0.20 μm; Agilent J&W, Santa Clara, CA, USA). The oven program started at 45 °C with a 4 min hold, followed by heating to 175 °C at 13 °C/min and maintaining this temperature for 27 min. The temperature was subsequently increased to 215 °C at 4 °C/min and kept constant for another 35 min. The injector temperature was set at 250 °C, and the injected sample volume was 1 μL. Identification of individual fatty acids was achieved by comparing their chromatographic retention times with those of certified standards. Quantitative results were obtained using peak-area normalization and expressed as the relative proportion (%) of each fatty acid in the total fatty acid fraction.

### 2.8. Quality Evaluation During Accelerated Storage

The oil samples were placed in sealed glass bottles and subjected to accelerated oxidation in a constant-temperature oven at 70 °C, following a slightly modified method of Sun et al. [19]. Each bottle contained 100 mL of oil with approximately 10 mL of headspace. The bottles were sealed under ambient air and kept protected from light in the oven. During the 7-day storage period, samples were collected on days 0, 1, 2, 3, 4, 5, 6, and 7 for the determination of acid value (AV), peroxide value (PV), and *p*-anisidine value (*p*-AV). Three independent replicates were prepared for each treatment.

#### 2.8.1. Acid Value (AV)

AV was measured according to the AOAC (2000) [20] procedure with slight modifications. An accurately weighed 5 g portion of the oil sample was transferred to an Erlenmeyer flask and mixed with 50 mL of neutral ethanol. The mixture was brought to boiling and maintained for 5 min, after which 1 mL of phenolphthalein indicator was added. The resulting solution was titrated with standardized 0.1 mol/L potassium hydroxide (KOH) until the titration endpoint was reached. The AV was subsequently calculated using the following equation:

(1) $$\mathrm{AV}=\frac{56.1\mathrm{VN}}{W}$$

*V* represents the titration volume (mL) of the 0.1 N potassium hydroxide standard solution, *N* is the concentration of potassium hydroxide (0.1 N), and *W* is the mass of the oil sample (g).

#### 2.8.2. Peroxide Value (PV)

PV was measured following the AOAC (2000) [20] procedure. A 5 g aliquot of oil was transferred into a 250 mL Erlenmeyer flask and dissolved by vigorous mixing with 30 mL of acetic acid–trichloromethane (3:2, *v*/*v*). Saturated KI solution (1.00 mL) was then introduced, and the flask was mixed thoroughly before being kept away from light for 3 min. Thereafter, 100 mL of distilled water was incorporated, and the liberated iodine was titrated with 0.01 mol/L standardized Na_2_S_2_O_3_ solution. As the yellow color approached disappearance, 1 mL of starch indicator was introduced, and titration was continued until the blue coloration was no longer visible. A reagent blank was analyzed concurrently using the same procedure to correct for background effects. The PV was obtained from the following equation:

(2) $$\mathrm{PV}=\frac{1000\times \left(V-V0\right)\times C}{M}$$

where *V* and *V*0 correspond to the volumes (mL) of sodium thiosulfate titrant required for the sample and blank determinations, respectively; *C* refers to the molar concentration of the sodium thiosulfate solution (0.01 mol/L); and *M* denotes the amount of oil used for analysis, expressed in grams.

#### 2.8.3. *p*-Anisidine Value (*p*-AV)

*p*-AV was determined according to the method described by Drinić et al., with minor modifications [21]. A 0.5 g portion of oil was dissolved in trimethylpentane and diluted with the same solvent to 25 mL. The initial absorbance was determined at 350 nm, with pure trimethylpentane serving as the reference. For color development, 5 mL of the prepared oil solution was added to 1 mL of *p*-aniline reagent containing 25 g/L *p*-aniline in glacial acetic acid. After thorough mixing, the reaction system was protected from light for 10 min. In parallel, a control was prepared by substituting trimethylpentane for the oil solution while keeping all other conditions unchanged. Following the reaction period, absorbance at 350 nm was recorded against the corresponding control.

### 2.9. Evaluation of Antioxidant Properties During Accelerated Storage

To evaluate oxidative changes and variations in antioxidant capacity during accelerated storage, DPPH and ABTS radical scavenging activities as well as ferric reducing antioxidant power (FRAP) were measured. Three independent replicates were prepared for each treatment.

#### 2.9.1. 2,2-Diphenyl-1-picrylhydrazyl (DPPH) and 2,2′-Azino-bis (3-Ethylbenzothiazoline-6-sulfonic Acid) (ABTS) Assay

The DPPH radical scavenging activity of the RO samples was determined based on the method of Uoonlue [22], with minor modifications. 100 mg of the RO sample was extracted with 1.5 mL of methanol under vigorous shaking. The extract was separated by centrifugation at 5000 rpm for 10 min. Subsequently, 150 μL of the methanolic extract was mixed with 150 μL of the DPPH working solution. The reaction mixture was vortex-mixed thoroughly and incubated in the dark at room temperature for 30 min, after which the absorbance was measured at 517 nm.

The ABTS radical scavenging activity of the RO samples was determined based on the method described by Uoonlue [22]. Approximately 100 mg of RO was extracted with 1.5 mL of methanol under vigorous shaking, and the mixture was centrifuged at 5000 rpm for 10 min. The ABTS radical cation was prepared by reacting 7 mmol/L ABTS in water with 2.45 mmol/L potassium persulfate at room temperature for 12–16 h. Before use, this stock solution was diluted with methanol until an absorbance of 0.70 ± 0.02 at 734 nm was achieved. A 100 μL aliquot of the methanolic oil extract was then combined with 1000 μL of the prepared ABTS working reagent and allowed to react for 5 min. The absorbance was subsequently determined at 734 nm.

The radical scavenging activities of DPPH and ABTS were calculated using Equation:

(3) Radical scavenging activity(%)=(1−Asample−Acontrol Ablank)×100

where *A*_sample_ is the absorbance of the sample reaction mixture, *A*_control_ is the absorbance of the sample control, and *A*_blank_ is the absorbance of the reagent blank.

Trolox was used as the reference antioxidant. The standard curves were described as follows:

For DPPH:

(4) $$y=2.8486x+0.7084(R^{2}=\;0.9991)$$

For ABTS:

(5) $$y=0.5042x-1.4213(R^{2}=\;0.9971)$$

where y represents radical scavenging activity (%) and x represents Trolox concentration (μg/mL). The antioxidant capacities of the samples were calculated from the corresponding calibration equations and expressed as (μg Trolox/mL).

#### 2.9.2. Ferric Reducing Antioxidant Power (FRAP) Assay

The FRAP of the RO samples was determined based on the method described by Lee [23], with modifications. The FRAP reagent was freshly formulated by mixing 20 mmol/L FeCl_3_·6H_2_O, 10 mmol/L TPTZ prepared in 40 mmol/L HCl, and 0.1 mol/L sodium acetate buffer at pH 3.6 in a 1:1:10 volume ratio. Approximately 100 mg of RO was extracted with 1 mL of 80% ethanol, 5 μL of the sample extract was mixed with 25 μL of distilled water and 170 μL of the FRAP working reagent. After incubation at 25 °C for 10 min, the absorbance was measured at 590 nm.

Trolox was used as the reference antioxidant. The calibration equation was

(6) $$\Delta A=0.0972x+0.0042(R^{2}=0.9997)$$

(7) FRAP (μmol Trolox/mL)=2.06×(ΔA−0.0042)

where ΔA represents the absorbance difference between the sample and blank, and x represents the amount of Trolox (nmol). The FRAP antioxidant capacity was calculated from the calibration equation and expressed as μmol Trolox/mL.

### 2.10. Determination of Lipid-Associated Bioactive Components During Accelerated Storage

This study investigated the effects of enzymatic degumming on the retention of lipid-soluble antioxidants and oxidative stability of RO during accelerated storage by monitoring dynamic changes in β-carotene, tocopherol, and phytosterols contents. Three independent replicates were prepared for each treatment.

#### 2.10.1. β-Carotene

β-Carotene was extracted from rapeseed oil according to Jiang et al. [24]. with slight modifications. 1.0 g of oil was extracted at 55 °C with shaking for 3 h using 60 mL of anhydrous ethanol as the extraction solvent, and the resulting extract was analyzed using a commercial plant β-carotene assay kit (FANKEW, Shanghai, China; catalog No. F0526-A) according to the manufacturer’s instructions. Absorbance was measured at 450 nm. A calibration curve was constructed using the β-carotene standards supplied with the kit, and the regression equation was y = 0.0026x − 0.0117 (R^2^ = 0.9981), where y represents the absorbance at 450 nm and x represents the β-carotene concentration (pg/mL). The β-carotene concentration of the samples was calculated from the calibration equation and corrected using the dilution factor (50-fold), extraction volume (60 mL), and sample mass (1.0 g). The final β-carotene content was expressed as mg/kg oil.

#### 2.10.2. Tocopherol

The tocopherol content was determined by HPLC according to the method described by Jiang [24]. For sample preparation, 1 g of the oil sample was accurately weighed, dissolved in n-hexane, and brought to a final volume of 10 mL. The prepared solution was kept under refrigerated conditions until analysis. For calibration, 1.000 g each of α-, γ-, and δ-tocopherol standards was separately dissolved in 10 mL of n-hexane to obtain individual stock solutions. Appropriate aliquots of these stock solutions were then diluted to yield working standards at 5, 20, 50, 100, 150, and 200 μg/mL, which were used to construct the corresponding calibration curves.

Tocopherols were quantified using a Waters E2695 HPLC instrument (Waters Corporation, Milford, MA, USA) coupled with a ZORBAX SB-C18 column (4.6 mm × 250 mm, 5 μm; Agilent Technologies, Santa Clara, CA, USA). Chromatographic separation was conducted under isocratic conditions with methanol–water (98:2, *v*/*v*) as the mobile phase at 1 mL/min. The column compartment was thermostated at 30 °C, and the injection volume was 20 μL. Eluted compounds were monitored with a diode-array detector at 295 nm, and each chromatographic run lasted 15 min. Standard curves were established by relating tocopherol concentration (x) to peak area response (y). The individual tocopherol species were identified according to the retention times of authentic reference compounds, and their amounts were calculated from the corresponding peak-area responses.

#### 2.10.3. Phytosterols

The phytosterol content was determined according to the method described by Wang [25], with appropriate modifications. For saponification, 0.5 g of the oil sample was mixed with 0.5 mL of 5α-cholestane internal standard solution (0.5 mg/mL) and 5 mL of 2 mol/L KOH in ethanol and heated at 85 °C for 1 h. Once the mixture had cooled to room temperature, 5 mL of distilled water was introduced, and the unsaponifiable components were isolated by extracting the mixture twice with n-hexane. The collected hexane fractions were combined and concentrated to dryness under a gentle nitrogen stream. The dry residue was subsequently re-dissolved and reacted with 100 μL of BSTFA–TMCS derivatization reagent prior to GC–MS determination.

Phytosterols were analyzed on an Agilent 7890A GC system interfaced with a 5975C mass-selective detector and equipped with a DB-5MS capillary column. The injector and transfer line were both maintained at 310 °C. Samples (2 μL) were introduced using a 2:1 split, with the carrier gas supplied at 1.5 mL/min. A solvent delay of 5 min was applied. The oven program began at 200 °C, which was held for 2 min, followed by heating to 315 °C at 25 °C/min; the overall run time was 13 min. Spectral acquisition was performed in full-scan mode across *m*/*z* 29–550. The quadrupole and ion source were operated at 150 and 250 °C, respectively, while electron ionization was conducted at 70 eV. Phytosterol species were assigned by comparison with the retention behavior of reference compounds. Phytosterol contents were quantified using 5α-cholestane as an internal standard based on the peak area ratios of individual phytosterols to the internal standard, and the results were expressed as mg/100 g oil.

### 2.11. Statistical Analysis

For each treatment, three independent experiments replicates were performed (n = 3), with one analytical measurement obtained for each parameter from each replicate. The three replicate values were used directly as the units of statistical analysis, and the data are presented as the mean ± standard deviation (SD). For the accelerated storage experiment, three independent replicates were analyzed for each treatment at each storage time. All statistical evaluations were conducted with IBM SPSS Statistics version 22.0 (SPSS Inc., Chicago, IL, USA). Differences among groups were assessed by one-way ANOVA followed by Fisher’s LSD post hoc test for pairwise comparisons, with values of *p* < 0.05 considered statistically significant. For the accelerated storage experiment, the effects of treatment, storage time, and their interaction (treatment × storage time) were evaluated using two-way ANOVA. Pairwise comparisons of estimated marginal means were conducted with Bonferroni adjustment to determine significant differences among treatments and storage times. Statistical significance was considered at *p* < 0.05.

## 3. Results

### 3.1. Optimization of the Enzymatic Degumming Process

#### 3.1.1. Single-Factor Optimization of the Degumming Process

**Figure 1:**
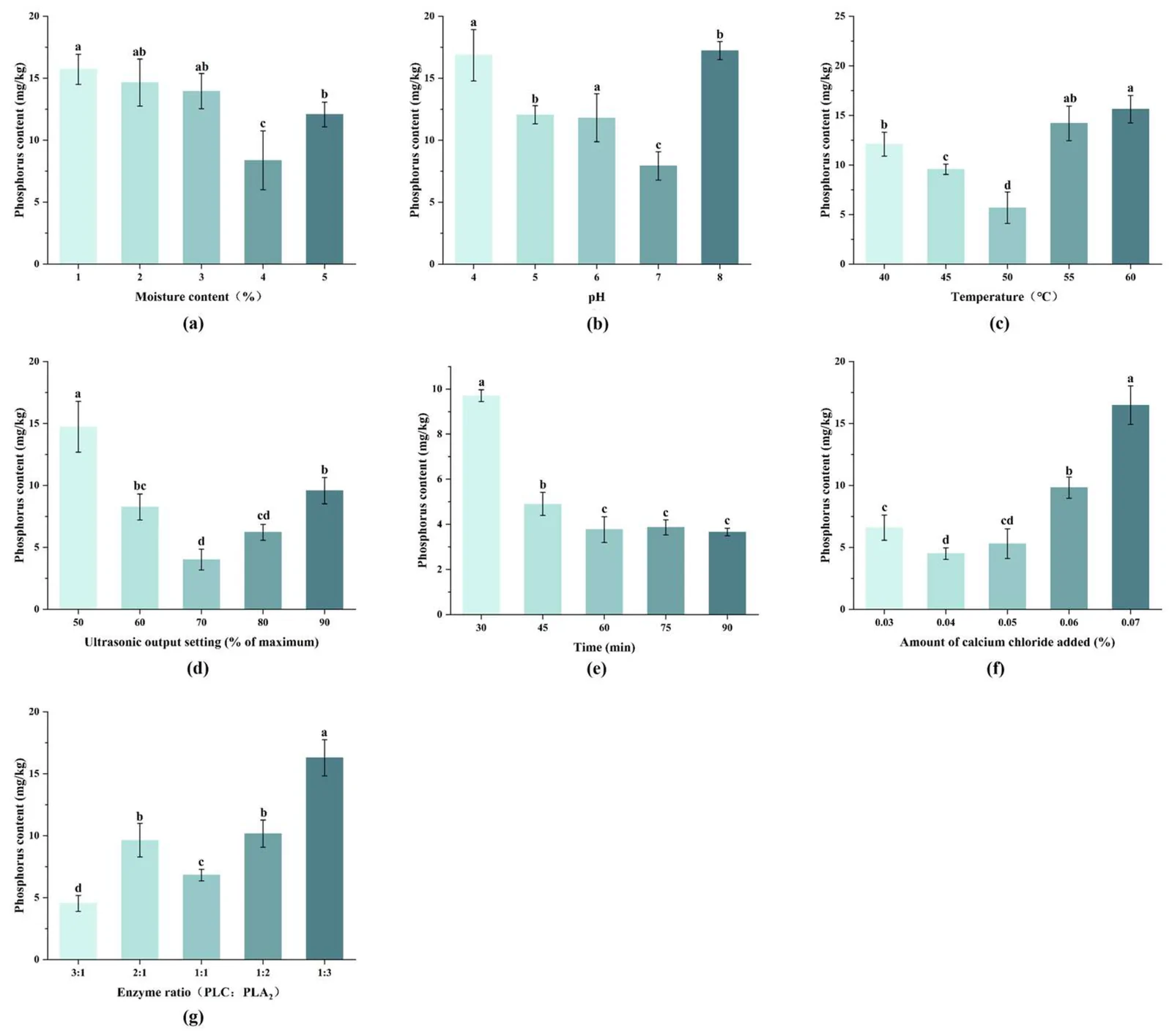
Optimization of enzymatic degumming process. (**a**) Effect of moisture content on reducing phospholipid content in RO: pH = 6, temperature = 50 °C, Ultrasonic output setting = 70%, ultrasound time = 45 min, CaCl_2_ addition = 0.07%, PLC: PLA_2_ = 1:1. (**b**) Effect of pH on reducing phosphorus content in RO: water addition = 4%, temperature = 50 °C, ultrasonic output setting = 70%, ultrasound time = 45 min, CaCl_2_ addition = 0.07%, PLC: PLA_2_ = 1:1. (**c**) Effect of temperature on reducing phosphorus content in RO: water addition = 4%, pH = 6, ultrasonic output setting = 70%, ultrasound time = 45 min, CaCl_2_ addition = 0.07%, PLC: PLA_2_ = 1:1. (**d**) Effect of ultrasonic output on reducing phosphorus content in RO: water addition = 4%, pH = 6, temperature = 50 °C, ultrasound time = 45 min, CaCl_2_ addition = 0.07%, PLC: PLA_2_ = 1:1. (**e**) Effect of time on reducing phosphorus content in RO: water addition = 4%, pH = 6, temperature = 50 °C, ultrasonic output setting = 70%, CaCl_2_ addition = 0.07%, PLC: PLA_2_ = 1:1. (**f**) Effect of CaCl_2_ addition on reducing phosphorus content in RO: water addition = 4%, pH = 6, temperature = 50 °C, ultrasonic output setting = 70%, ultrasound time = 60 min, PLC: PLA_2_ = 1:1. (**g**) Effect of enzyme ratio on reducing phosphorus content in RO: water addition = 4%, pH = 6, temperature = 50 °C, ultrasonic output setting = 70%, ultrasound time = 60 min, CaCl_2_ addition = 0.04%. Results are expressed as the mean ± SD of three independent experiments (*n* = 3). Different letters indicate significant differences among treatments (*p* < 0.05).

As shown in Figure 1a, the residual phosphorus content in RO gradually decreased as the moisture addition increased from 1% to 4%, reaching a minimum at 4%. However, when moisture was further increased to 5%, the residual phosphorus content increased. This trend indicates that an appropriate moisture level facilitates phospholipid hydration and enhances the interfacial contact between PLC and PLA_2_ and phospholipid substrates at the oil–water interface [26], thereby improving enzymatic degumming efficiency. In contrast, excessive moisture may intensify emulsification under ultrasonic conditions [17], hinder oil–water separation, and consequently reduce degumming performance. Therefore, a moisture addition of 4% was selected for subsequent experiments.

As shown in Figure 1b, the residual phosphorus content in RO gradually decreased as the pH increased from 4.0 to 7.0, reaching a minimum at pH 7.0. When the pH was further increased to 8.0, the residual phosphorus content increased again, indicating that excessively acidic and alkaline conditions were unfavorable for enzymatic degumming. An appropriate pH helps maintain the conformation and catalytic activity of phospholipase [26], while also influencing the ionization state and interfacial distribution of phospholipid substrates [27], thereby facilitating enzyme–substrate interactions. Therefore, a pH of 7.0 was selected for subsequent experiments.

As shown in Figure 1c, when the reaction temperature increased from 40 °C to 50 °C, the residual phosphorus content in RO decreased significantly and reached a minimum at 50 °C (*p* < 0.05). Further increases in temperature led to a gradual rise in residual phosphorus content. Moderate temperature elevation reduces oil viscosity, thereby promoting the diffusion of phospholipids and enhancing their contact with phospholipase at the oil–water interface [17], which improves degumming efficiency. However, excessively high temperatures may reduce enzyme activity or disrupt its conformational stability [28,29], thereby weakening phospholipid hydrolysis. Therefore, the reaction temperature was set at 50 °C for subsequent experiments.

As shown in Figure 1d, the residual phosphorus content in RO progressively decreased as the ultrasonic output increased from 50% to 70% of the maximum, but increased again when the output was further raised to 80% and 90%. Moderate increases in ultrasonic power enhance cavitation intensity and system turbulence. This, in turn, enlarges the oil–water interfacial area and promotes contact between phospholipase and phospholipid substrates [30], thereby improving degumming efficiency. In contrast, excessively high ultrasonic power may intensify emulsification or impair enzyme conformational stability [31,32], thereby reducing degumming effectiveness. Therefore, an ultrasonic output of 70% of the maximum was selected for subsequent experiments.

As shown in Figure 1e, one-way ANOVA followed by the LSD post hoc test showed that the residual phosphorus content in RO significantly decreased as the ultrasound time increased from 30 to 60 min (*p* < 0.05). In contrast, no significant differences were observed between the 60 and 75 min treatments (*p* = 0.778) or between the 60 and 90 min treatments (*p* = 0.748), indicating that the dephosphorization efficiency had reached a stable plateau. Therefore, an ultrasound time of 60 min was selected for subsequent experiments, as further prolongation of the treatment did not significantly reduce the residual phosphorus content.

As shown in Figure 1f, the residual phosphorus content in RO initially decreased and then increased with increasing CaCl_2_ addition, reaching a minimum at 0.04% (*w*/*w*, based on oil weight). A moderate amount of Ca^2+^ may help maintain PLA_2_ catalytic activity and promote colloidal particle aggregation [33], thereby enhancing degumming efficiency. In contrast, excessive Ca^2+^ may increase emulsion stability [34], hinder gum aggregate formation, and impede centrifugal separation, leading to higher residual phosphorus content. Therefore, a CaCl_2_ addition level of 0.04% (*w*/*w*, based on oil weight) was selected for subsequent experiments.

As shown in Figure 1g, the residual phosphorus content was highest when PLC: PLA_2_ ratio was 1:3. As the proportion of PLC increased, the residual phosphorus content gradually decreased, reaching a minimum at a ratio of 3:1. This may be attributed to the efficient hydrolysis of major phospholipids such as phosphatidylcholine (PC) and phosphatidylethanolamine (PE) by PLC, generating diacylglycerol and water-soluble phosphorylated head groups [35], thereby facilitating phospholipid removal. Additionally, an appropriate amount of PLA_2_ can further hydrolyze the sn-2 acyl bonds of residual phospholipids, enhancing their separability [36]. However, when the PLA_2_ proportion is too high, the PLC activity becomes insufficient, and the resulting lysophospholipids may increase emulsion stability, thereby hindering phospholipid removal. Passos et al. also reported that PLC effectively removes PC and PE, and that a combined phospholipase system helps reduce residual phosphorus levels [37]. Therefore, the optimal PLC: PLA_2_ ratio was determined to be 3:1 for subsequent experiments.

The single-factor results indicated that moisture content, pH, and temperature influenced the residual phosphorus content in oil, with each factor exhibiting an apparent optimal range. Previous studies have also identified moisture content, pH, and temperature as important operational parameters affecting enzymatic degumming efficiency. Water addition promotes phospholipid hydration and facilitates enzyme–substrate interactions [30,37], whereas temperature and pH regulate phospholipase activity and substrate hydrolysis efficiency, thereby affecting the overall degumming performance [17,29]. Based on these experimental observations and supporting evidence from previous studies, these three parameters were selected as independent variables for subsequent optimization using response surface methodology (RSM). A Box–Behnken design was then applied to evaluate the effects of moisture content, pH, and temperature on residual phosphorus content, which was selected as the response variable. The remaining factors were maintained at their optimal levels determined from the single-factor experiments.

#### 3.1.2. RSM Analysis

Based on the single-factor results, A Box–Behnken design combined with response surface methodology (RSM) was subsequently applied to refine the process conditions. The analysis of variance of the quadratic model (Table 1) showed an F-value of 83.49 (*p* < 0.0001), confirming the significance of the regression model. The lack-of-fit term was not significant (*p* = 0.5066), indicating good model adequacy. The coefficient of determination (R^2^ = 0.9908) demonstrated an excellent fit between the model and experimental data, indicating strong predictive capability. The model exhibited an adequate precision value of 25.61, representing its signal-to-noise ratio. Because this value was well above the commonly accepted minimum of 4, the model was considered to have sufficient predictive reliability for exploration within the experimental design space.

**Figure 2:**
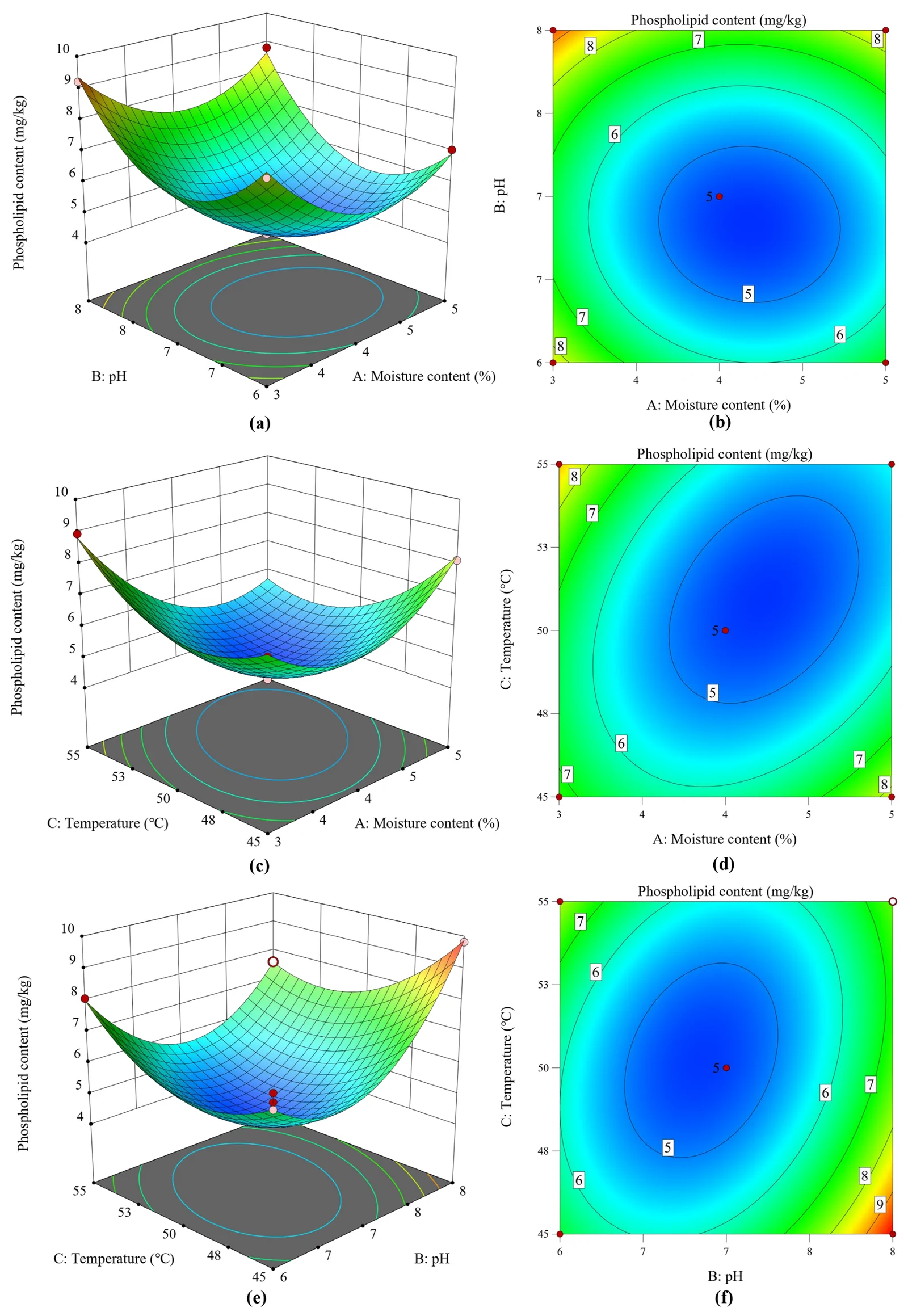
Three-dimensional surface and two-dimensional contour plots of response surface methodology (RSM): (**a**,**b**) Relationship between moisture content and pH; (**c**,**d**) Relationship between moisture content and temperature; (**e**,**f**) Relationship between pH and temperature. The color gradient from blue to green and yellow represents increasing predicted response values. The colored dots denote the experimental design points; red dots indicate observed response values higher than the model-predicted values, whereas pale pink dots indicate observed response values lower than the predicted values.

Figure 2a,c,e present the response surface plots, Consistent with these response surface plots, the ANOVA results showed significant interactions between A and C (*p* = 0.0001) and between B and C (*p* = 0.0003), whereas the interaction between A and B was not significant (*p* = 0.2218). In Figure 2b, the elliptical contour lines further confirm the significant interaction between A and C, consistent with the ANOVA results. Figure 2d,f indicate a significant interaction between B and C, whereas the interaction between A and B is not significant. Based on response surface optimization, the optimal conditions for enzymatic degumming of RO were determined as follows: moisture content of 4%, pH 7, and temperature of 51 °C. Verification experiments conducted under these conditions yielded a phosphorus content of 4.55 mg/kg, which closely matched the model prediction (4.52 mg/kg), confirming the reliability of the established response surface model.

### 3.2. Phospholipid Content Analysis

**Figure 3:**
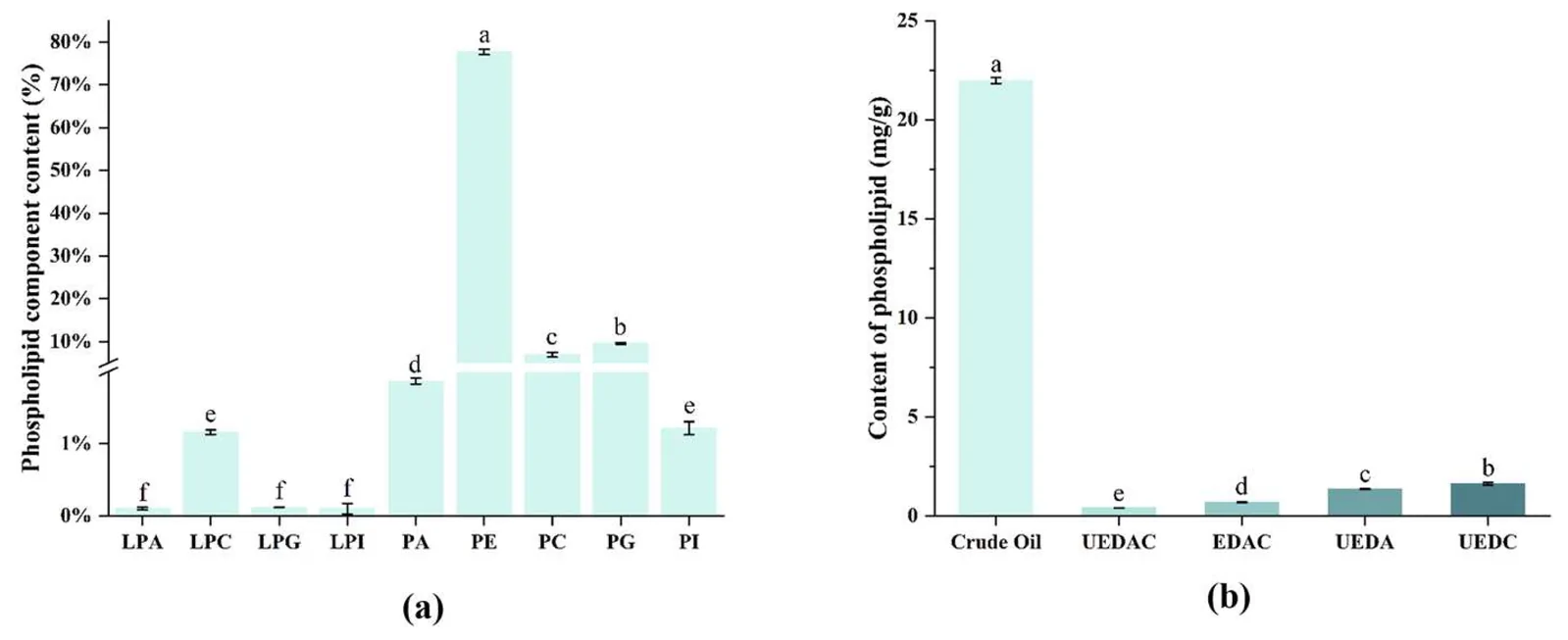
(**a**) Phospholipid composition in crude RO; (**b**) phospholipid content in RO after different degumming processes. Results are expressed as the mean ± SD of three independent experiments (*n* = 3). Different lowercase letters indicate significant differences among groups within each panel according to Fisher’s LSD post hoc test (*p* < 0.05). One-way ANOVA revealed significant differences among phospholipid classes in panel (**a**) and a significant treatment effect in panel (**b**) (*p* < 0.001). Abbreviations: Crude oil: untreated RO; UEDAC: ultrasound-assisted PLC/PLA_2_ composite enzyme degumming; EDAC: non-ultrasound PLC/PLA_2_ composite enzyme degumming; UEDA: ultrasound-assisted PLA_2_ degumming; UEDC: ultrasound-assisted PLC degumming.

As shown in Figure 3a, nine phospholipid species were identified in crude RO, among which PE, PC, and PG were the dominant classes, all belonging to glycerophospholipids (GPs), consistent with previous reports [38]. PE was the most abundant component, while lysophosphatidic acid (LPA), lysophosphatidylglycerol (LPG), and lysophosphatidylinositol (LPI) were present at lower levels. Significant differences were observed among the phospholipid classes (F(8, 18) = 22,264.316, *p* < 0.001). To further evaluate degumming efficiency, the total phospholipid content of RO obtained under different treatments was compared. As shown in Figure 3b, the total phospholipid content of crude RO was 21.98 ± 0.15 mg/g, Degumming treatment significantly affected phospholipid content (F(4, 10) = 44,234.646, *p* < 0.001), and all degummed samples showed significantly lower values than crude RO (*p* < 0.05). Among the degumming treatments, UEDAC exhibited the lowest residual phospholipid content and achieved a removal rate of 98.19%. The residual phospholipid content of UEDAC was significantly lower than that of EDAC, UEDA and UEDC (*p* < 0.05). These results indicate that ultrasound-assisted PLC/PLA_2_ composite enzymatic degumming provides superior dephosphorization performance compared with other methods. Therefore, UEDAC can serve as an effective strategy for improving RO degumming efficiency.

### 3.3. Lipidomics Results

**Figure 4:**
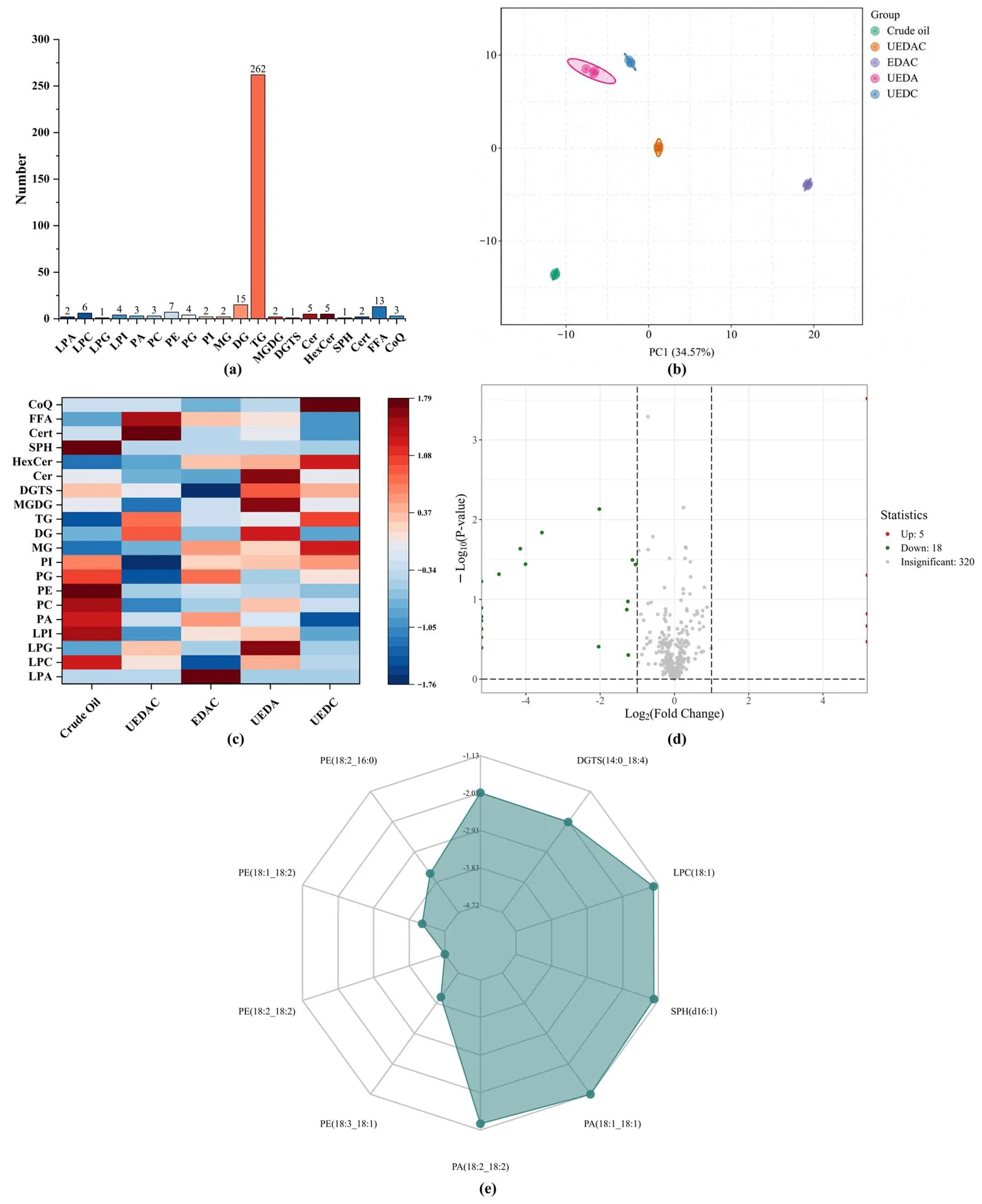
Lipidomic profiles of RO after enzymatic degumming. (**a**) lipid subclass and subclass identification quantity diagram; (**b**) Principal component analysis of mass spectrometry data; (**c**) Hierarchical clustering heatmap of differentially metabolized compounds; (**d**) Volcano plot of differences between crude oil and UEDAC; (**e**) Radar plot comparing crude oil and UEDAC. Differential lipids were identified using a fold change (FC) ≥2 or ≤0.5.

Lipidomics has been widely applied in vegetable oil processing, as it enables systematic characterization of lipid compositional changes under different processing conditions [39]. As shown in Figure 4a, a total of 340 lipid species were identified and classified into 19 lipid subclasses. Based on lipid categories, these compounds mainly included glycerolipids (GLs, 282 species), glycerophospholipids (GPs, 28 species), fatty acyls (FAs, 13 species), sphingolipids (SPs, 12 species), and other lipids (5 species). Among them, triacylglycerols (TGs) represented the most abundant subclass, with 262 species identified and accounting for the largest proportion of total lipid molecules. As shown in Figure 4b, PCA demonstrated that the first two principal components explained 34.57% and 22.42% of the overall variation, respectively, accounting for 56.99% in combination. A clear distinction was observed between the crude RO samples and all degummed samples. In addition, the UEDA, UEDC, UEDAC, and EDAC treatments were distributed in separate regions of the score space. This group-specific clustering suggests that the different degumming strategies induced distinct alterations in the lipid composition of RO.

As shown in Figure 4c, crude RO and the different degummed oil samples exhibited distinct clustering patterns based on the relative abundance of lipid subclasses, indicating that different degumming processes altered the lipid profile of RO to varying extents. Compared with crude oil, the relative abundances of major phospholipid subclasses, including PC, PE, PI, and PG, generally decreased after degumming, suggesting that enzymatic degumming effectively removed phosphorus-containing gum components from the oil. This finding is consistent with the hydrolysis, migration, and phase separation of phospholipids during the degumming process. Differences in lipid profiles were also observed among the various degumming treatments [12]. In particular, the relative abundance of DG increased in the UEDC group, which may be attributed to the hydrolysis of phosphodiester bonds in phospholipids by PLC, leading to the formation of DGs. In contrast, PLA_2_ mainly hydrolyzes the ester bond at the sn-2 position of phospholipids, producing lysophospholipids and free fatty acids; therefore, changes in lysophospholipid-related components were observed in the UEDA group.

To further investigate the lipidomic alterations induced by degumming, differential lipid analysis was performed between crude RO and UEDAC. As shown in Figure 4d, based on the differential screening criterion (FC ≥ 2 or ≤ 0.5), a total of 23 lipid molecular species were significantly altered during the degumming process. Based on the magnitude of log_2_ FC, the ten most markedly changed lipid species were selected for further analysis (Figure 4e). All ten lipid species showed decreasing trends after degumming, including PE(18:2_16:0), PE(18:1_18:2), PE(18:2_18:2), PE(18:3_18:1), LPI(16:0), LPC(18:1), PA(18:1_18:1), PA(18:2_18:2), DGTS(14:0_18:4), and SPH(d16:1). Notably, several PE species exhibited pronounced decreases, indicating that PE was one of the phospholipid subclasses most strongly affected during RO degumming. PE is generally regarded as an important non-hydratable phospholipid in crude vegetable oils [40]. Its ethanolamine head group can interact with metal ions to form phospholipid–metal complexes, which may remain in the oil phase even after conventional hydration degumming. During enzymatic degumming, PLC hydrolyzes the phosphodiester bond of susceptible PE molecules, yielding DAG and a water-soluble phosphorylated head group [41], whereas PLA_2_ hydrolyzes the acyl ester bond at the sn-2 position of phospholipids to generate LPE and FFAs [42]. Therefore, the combined PLC–PLA_2_ system provides two complementary hydrolytic pathways for the conversion and removal of residual PE, which may account for the pronounced decreases observed in multiple PE molecular species after UEDAC treatment.

**Figure 5:**
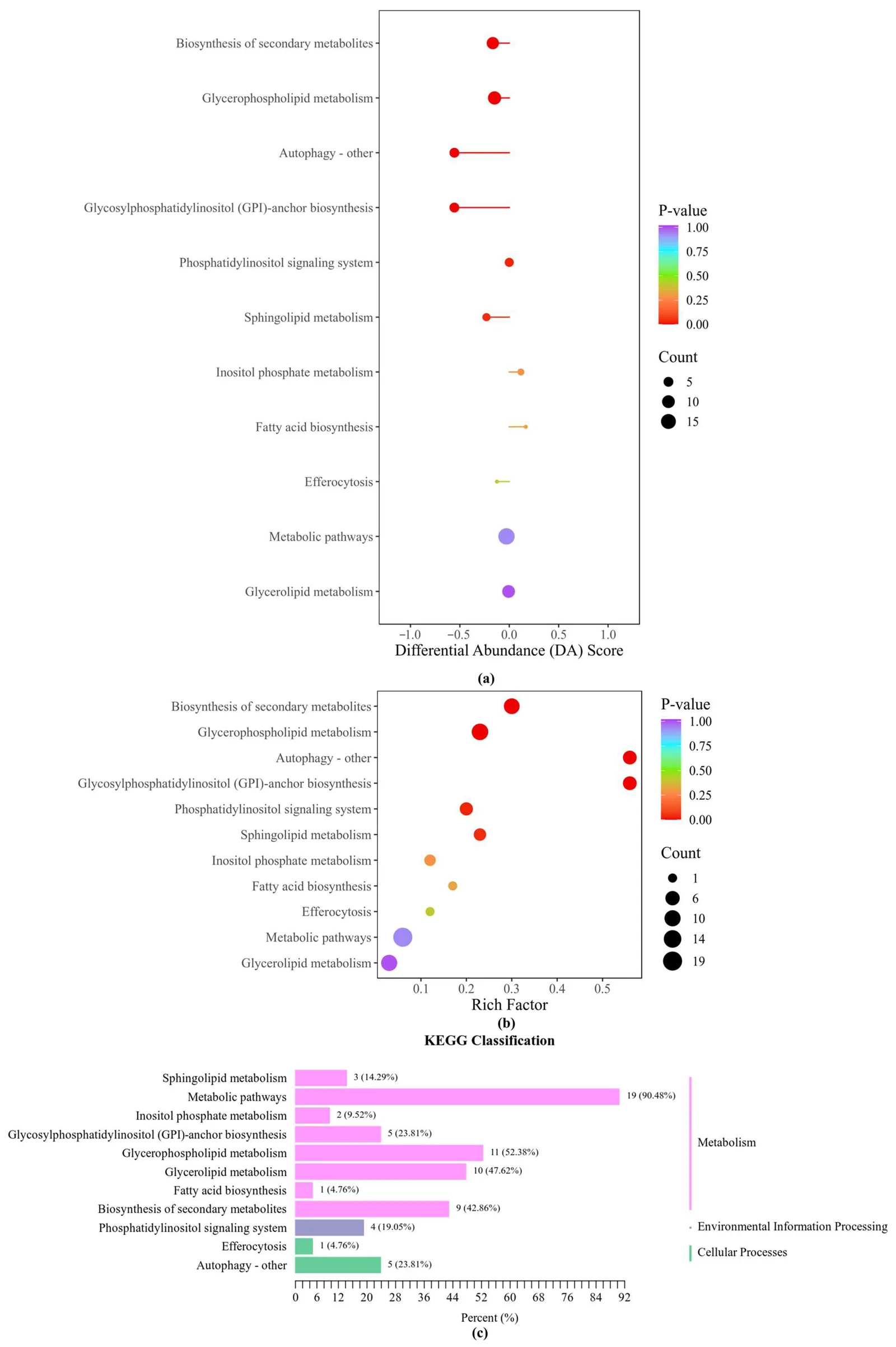
KEGG pathway analysis between crude oil and UEDAC. (**a**) Differential abundance scores of metabolic pathways; (**b**) Bubble plot of KEGG pathway enrichment for differential lipids; (**c**) KEGG pathway classification analysis of differential lipids. The Rich factor represents the proportion of differential lipids in each pathway, with dot size indicating the number of differential lipids and dot color indicating the enrichment *p*-value. The DA score reflects the overall direction of lipid changes within each pathway, with positive and negative values indicating increasing and decreasing trends, respectively.

The differential abundance score reflects the overall trend of changes in differentially abundant metabolites within specific pathways, ranging from −1 to 1. As shown in Figure 5a, significant alterations were observed in glycerophospholipid metabolism, sphingolipid metabolism, glycerolipid metabolism, and related pathways. This indicates that UEDAC treatment primarily affects GPs and GLs compared with crude oil. KEGG enrichment analysis further revealed that glycerophospholipid metabolism contained a higher number of differentially abundant lipids and exhibited lower *p*-values, suggesting it is one of the pathways most closely associated with UEDAC treatment (Figure 5b). KEGG classification results showed that differentially abundant lipids were mainly involved in metabolic pathways, glycerophospholipid metabolism, glycerolipid metabolism, and secondary metabolite biosynthesis, accounting for 90.48%, 52.38%, 47.62%, and 42.86%, respectively (Figure 5c). Changes in glycerophospholipid metabolism indicated extensive remodeling of phosphorus-containing lipids in RO during the treatment process. The combined action of the two phospholipases promoted the simultaneous hydrolysis and conversion of phospholipids, thereby facilitating the separation and removal of residual phospholipids, which was consistent with the changes described above. On the other hand, glycerolipid metabolism was also an important pathway affected by enzymatic degumming, and its alteration may be closely associated with the formation of DAG through PLC-mediated phospholipid hydrolysis. Overall, the lipidomics profile of the UEDAC treatment indicates that ultrasound-assisted PLC/PLA_2_ composite enzyme degumming primarily influences the degradation of GPs and modulates the composition of GLs during RO processing. These findings further confirm the advantages of UEDAC in enhancing RO degumming efficiency.

### 3.4. Changes in Metal Ion Contents

Metal ion content can indirectly reflect the level of gums in RO, and its reduction is also beneficial for decreasing the risk of emulsification and refining losses during subsequent processing [13]. As shown in Table 2, the Mg and Ca contents in crude RO were 53.68 mg/kg and 166.15 mg/kg, respectively. Both were significantly decreased after different degumming treatments (*p* < 0.05), indicating that enzymatic degumming effectively promoted the removal of metal ions. Among all treatments, the UEDAC group exhibited the lowest Ca content (22.06 mg/kg). The lowest Mg content was observed in the UEDA group (2.57 mg/kg), while that in the UEDAC group was 3.28 mg/kg. These differences may be attributed to the substrate specificity of different phospholipases and the enhanced mass transfer at the oil–water interface induced by ultrasonic treatment. Ye et al. [43] reported that PLC mainly acts on phospholipids such as PC and PE, whereas its hydrolytic activity toward non-hydratable phospholipids such as PI and PA, which are strongly associated with Ca and Mg, is relatively limited. This may explain the higher residual metal ion levels observed in the UEDC group. In contrast, PLC and PLA_2_ in the UEDAC system may exert complementary effects. Specifically, PLC preferentially hydrolyzes phospholipids such as PC and PE, whereas PLA_2_ acts on certain components associated with non-hydratable phospholipids, including PI and PA [44], thereby promoting the transformation and removal of metal ion-bound phospholipids. In addition, UEDAC ultrasound treatment can enhance oil–water dispersion and improve the contact efficiency between enzymes and substrates, thereby further increasing the removal efficiency of metal ions.

### 3.5. Fatty Acid Analysis

As shown in Table 3, after different enzymatic degumming treatments, a total of 13 fatty acids were identified across the crude and degummed RO samples, mainly including palmitic acid (C16:0), oleic acid (C18:1), linoleic acid (C18:2), and alpha-linolenic acid (C18:3). Among them, oleic acid (C18:1) showed the highest content, ranging from 50.92% to 55.19%, followed by linoleic acid (C18:2), which ranged from 25.60% to 27.84%. These results indicate that RO is mainly composed of unsaturated fatty acids (UFA, 85.27–89.09%), consistent with previous reports on the fatty acid composition of RO [43]. Crude oil and EDAC exhibited comparable UFA proportions (89.09% and 88.93%, respectively), whereas UEDAC showed a significant decrease to 87.40% (*p* < 0.05). The lowest UFA proportions were observed in UEDA (85.27%) and UEDC (85.39%), which did not differ significantly from each other but were significantly lower than those in the other treatments (*p* < 0.05). Among them, the content of monounsaturated fatty acids (MUFAs) ranged from 53.84% to 58.21%, while that of polyunsaturated fatty acids (PUFAs) ranged from 28.72% to 31.92%, overall showing the pattern MUFA > PUFA > SFA. A higher proportion of MUFA and PUFA can enhance the nutritional value of RO [45]. The lower SFA content is consistent with the characteristics of healthy edible oils and may help reduce dietary saturated fatty acid intake and the risk of cardiovascular diseases [24]. Notably, decanoic acid (C10:0) was detected in the ultrasound-assisted treatments but was not detected in crude oil or EDAC. This may be associated with ultrasound-induced lipid oxidation, as ultrasonic cavitation has been reported to accelerate oil oxidation [17]. Oxidation of long-chain UFA such as oleic acid can involve hydroperoxide formation and subsequent oxidative cleavage, and C10:0 has been identified among the oxidation products of oleic acid [46,47].

### 3.6. Analysis of Quality Changes During Accelerated Storage

**Figure 6:**
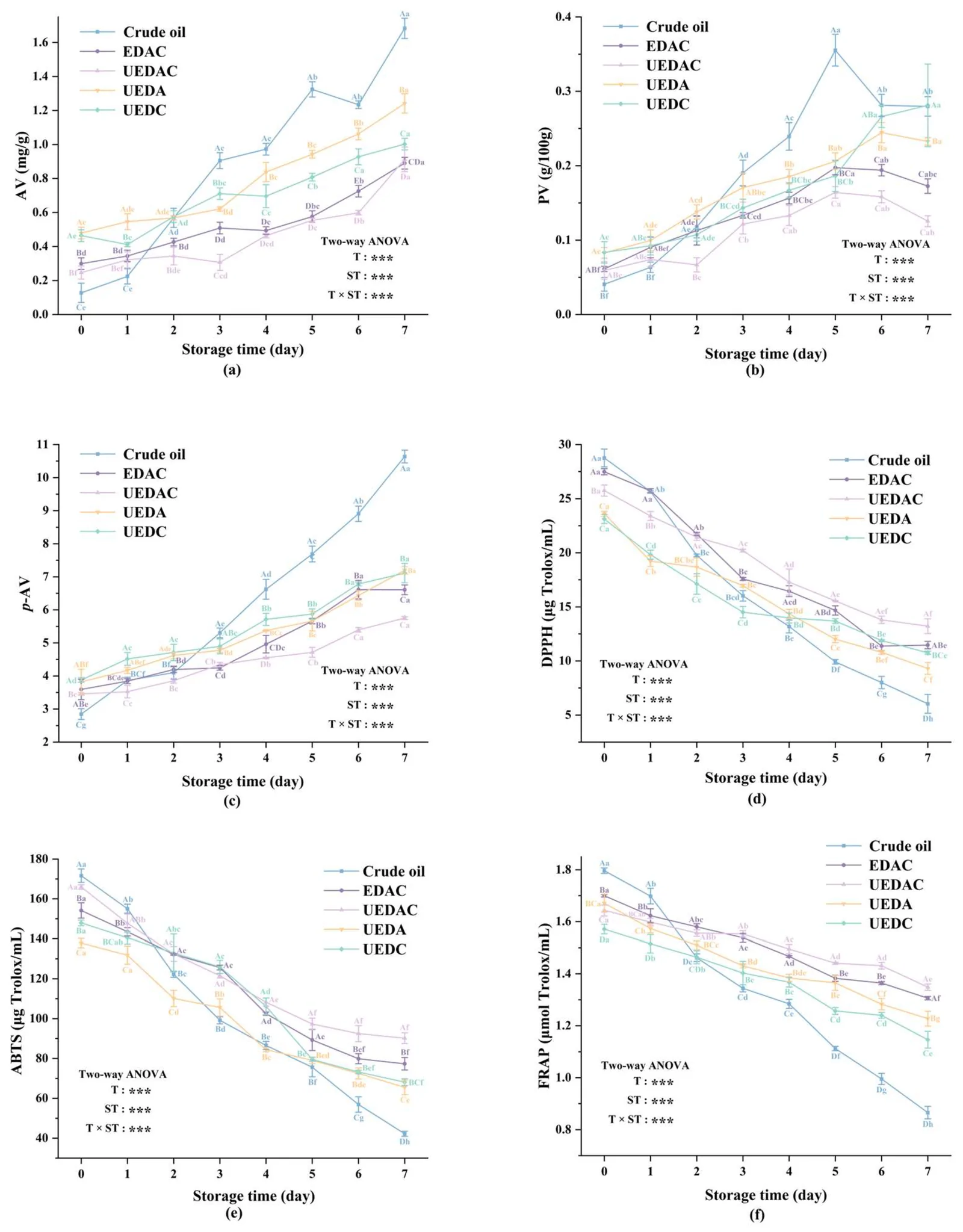
Changes in quality and antioxidant capacity during accelerated storage under different processing methods. (**a**) AV; (**b**) PV; (**c**) *p*-AV; (**d**) DPPH; (**e**) ABTS; (**f**) FRAP. T, ST, and T × ST represent the effects of treatment, storage time, and their interaction, respectively, in the two-way ANOVA. Pairwise comparisons were adjusted using the Bonferroni method. Results are expressed as the mean ± SD of three independent experimental. Different uppercase letters indicate significant differences among treatments at the same storage time, whereas different lowercase letters indicate significant differences among storage times within the same treatment (*p* < 0.05). Asterisks indicate the significance levels of two-way ANOVA (* *p* < 0.05, ** *p* < 0.01, and *** *p* < 0.001).

Figure 6 shows the changes in the physicochemical and antioxidant properties of crude and degummed RO during the 7-day accelerated storage test at 70 °C. Two-way ANOVA revealed significant effects of treatment, storage time, and their interaction on all evaluated parameters (all *p* < 0.001), indicating that the effects of the treatments varied over the storage period.

#### 3.6.1. Acid Value (AV) Analysis

AV is an important indicator for evaluating the extent of hydrolytic rancidity in oils and fats [37]. As shown in Figure 6a, during the 7-day accelerated storage test at 70 °C, the acid values (AVs) of all groups increased to varying degrees with prolonged storage time, indicating a gradual intensification of hydrolytic rancidity under accelerated storage conditions. On day 0, the AV of all degummed RO samples were higher than that of crude RO (0.12 mg/g), which may be associated with changes occurring during the degumming process. In particular, in the UEDA group, PLA_2_ can hydrolyze phospholipid acyl groups, thereby releasing a certain amount of free fatty acids [48]. In contrast, starting from day 3, the acid values of all enzyme-treated samples were lower than that of untreated crude RO. This trend suggests that, although the degumming treatment temporarily increased AV, the degummed oils exhibited better stability during subsequent accelerated storage. Especially in the UEDAC group, the increase in AV was minimal, demonstrating superior control of acid value. This observation may be associated with the removal of phospholipids, metal ions, and other pro-rancidity impurities under the applied processing conditions. Overall, these results indicate that UEDAC exhibited better hydrolytic stability than crude RO under the tested accelerated storage conditions.

#### 3.6.2. Peroxide Value (PV) Analysis

PV is a classic indicator of primary oxidation products in oils and fats, and it generally increases during storage. As shown in Figure 6b, crude RO showed the lowest initial PV (0.041 g/100 g), whereas slightly higher PV were observed after degumming, consistent with previous reports [32]. The EDAC and UEDAC groups maintained relatively lower PV levels during the middle and later stages of accelerated storage. Furthermore, compared with the EDAC group, the UEDAC group consistently showed lower PV values during the accelerated storage test. This suggests that ultrasound assistance further enhances the removal of gums and other pro-oxidative components during PLC/PLA_2_ composite enzymatic degumming. As a result, the formation and accumulation of primary oxidation products were effectively suppressed under accelerated storage conditions. Notably, PV decreased or fluctuated in some groups during the later stage of accelerated storage, which may be associated with the instability of peroxides and their subsequent decomposition into secondary oxidation products, including aldehydes and ketones [49]. Therefore, PV variation should be interpreted in combination with secondary oxidation indicators for a more comprehensive evaluation of lipid oxidation behavior.

#### 3.6.3. *p*-Anisidine Value (*p*-AV) Analysis

*p*-AV is used to evaluate the accumulation of secondary oxidation products in RO [50]. As shown in Figure 6c, on day 0, the *p*-AV of crude RO was lower than that of degummed RO in all treatment groups. This may be attributed to heating, stirring, ultrasonic treatment, and exposure to oxygen during the degumming process, which promoted the conversion of part of the hydroperoxides into secondary oxidation products. During the accelerated storage test, *p*-AV values in all groups gradually increased, indicating continuous lipid oxidation under accelerated storage conditions, accompanied by ongoing decomposition of hydroperoxides and the formation of secondary oxidation products such as aldehydes and ketones. Notably, the *p*-AV of RO in the UEDAC group remained at a relatively low level during the accelerated storage test, suggesting that this treatment effectively suppressed the accumulation of secondary oxidation products.

### 3.7. Analysis of Changes in Antioxidant Properties During Accelerated Storage

RO is prone to flavor deterioration, color darkening, and quality degradation during processing and storage; therefore, antioxidant capacity is an important indicator for evaluating its quality stability [51]. In this study, DPPH, ABTS, and FRAP assays were employed to comprehensively assess changes in the antioxidant capacity of enzymatically degummed RO during the accelerated storage test. As shown in Figure 6d–f, the DPPH and ABTS radical scavenging capacities, as well as the FRAP reducing antioxidant capacity, showed a continuous decreasing trend in all samples during the accelerated storage test. This indicates that endogenous antioxidants, such as tocopherols, may be gradually consumed under accelerated storage conditions, leading to a progressive decline in antioxidant capacity. At the initial stage of the accelerated storage test, crude oil exhibited the highest antioxidant capacity among all groups, with DPPH, ABTS, and FRAP values of 28.76 μg Trolox/mL, 171.66 μg Trolox/mL, and 1.80 μmol Trolox/mL, respectively. This may be attributed to the higher retention of natural antioxidant compounds in crude oil [52]. However, as the accelerated storage test progressed, the antioxidant capacity of crude oil decreased markedly. In contrast, the UEDAC group consistently maintained relatively higher DPPH, ABTS, and FRAP values throughout the accelerated storage test. These results suggest that, under optimized conditions, the beneficial effects of ultrasound-induced cavitation in enhancing degumming efficiency, promoting gum removal, and reducing pro-oxidant factors may play a dominant role. In addition, the synergistic action of PLC and PLA_2_ may contribute to more complete phospholipid hydrolysis and improved removal efficiency, thereby weakening pro-oxidant interactions among residual phospholipids, metal ions, and lipid hydroperoxides. This further inhibits the accumulation of lipid hydroperoxides and the formation of secondary oxidation products, such as aldehydes and ketones. Overall, UEDAC-treated RO showed better preservation of antioxidant capacity during accelerated storage.

### 3.8. Analysis of Changes in Bioactive Components During Accelerated Storage

**Figure 7:**
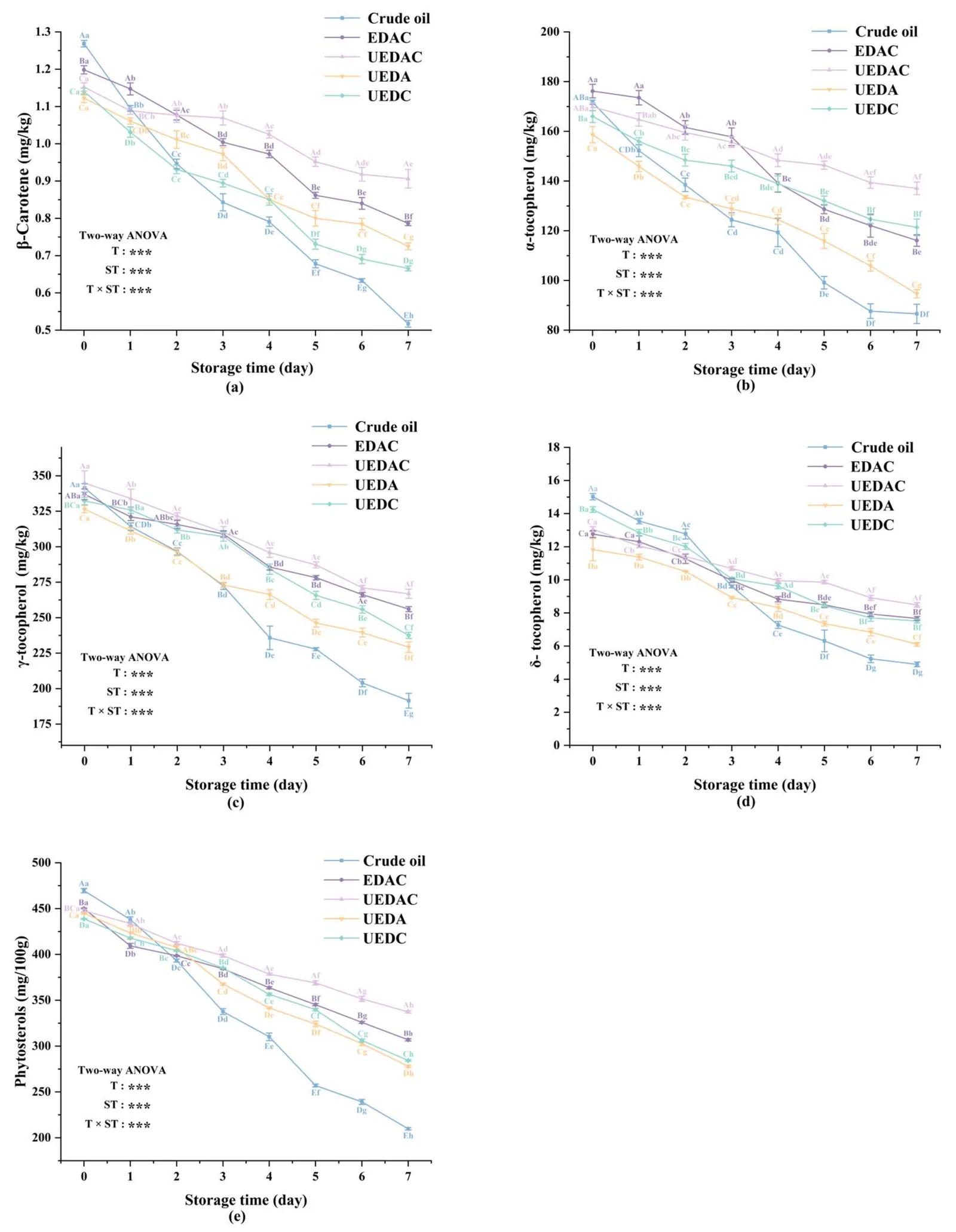
Changes in bioactive components during accelerated storage under different processing methods. (**a**) β-carotene; (**b**–**d**) three types of tocopherols; (**e**) phytosterols. T, ST, and T × ST represent treatment, storage time, and their interaction, respectively. Pairwise comparisons were Bonferroni-adjusted. Results are expressed as the mean ± SD of three independent experimental Different uppercase letters indicate significant differences among treatments at the same storage time, whereas different lowercase letters indicate significant differences among storage times within the same treatment (*p* < 0.05). Asterisks indicate the significance levels of two-way ANOVA (* *p* < 0.05, ** *p* < 0.01, and *** *p* < 0.001).

Figure 7 shows the changes in bioactive components of crude and degummed RO during accelerated storage. Two-way ANOVA showed significant effects of treatment, storage time, and their interaction on all bioactive components (all *p* < 0.001).

#### 3.8.1. β-Carotene Analysis

β-carotene is an important lipid-soluble bioactive component in RO, which can scavenge singlet oxygen and also contributes to the natural color of the oil [53]. As shown in Figure 7a, the β-carotene content in all treated RO samples decreased over time during the accelerated storage test, indicating varying degrees of oxidative degradation under accelerated storage conditions. On day 0, crude oil exhibited the highest β-carotene content (1.27 mg/kg), exceeding that of all enzyme-degummed samples that some initial loss occurred during the degumming process. This may be attributed to ultrasonic cavitation enhancing oxidative reactions in the oil system, thereby accelerating the degradation of oxygen-sensitive β-carotene [54]. In addition, β-carotene may be partially removed through adsorption onto the gum phase during degumming. By day 3 of the accelerated storage test, the β-carotene levels in all enzyme-degummed samples surpassed those of crude oil. Notably, the UEDAC group maintained consistently higher β-carotene levels from day 3 onward. This may be attributed to more effective removal of gums, metal ion complexes, and other pro-oxidative polar impurities by phospholipase-based degumming, thereby reducing their catalytic effects on β-carotene degradation under accelerated storage conditions. Overall, although UEDAC processing resulted in an initial limited loss of β-carotene, the UEDAC-treated RO showed better β-carotene retention during accelerated storage.

#### 3.8.2. Tocopherols Analysis

Tocopherols are important endogenous antioxidants in vegetable oils, and their content is closely associated with the oxidative stability of the oil system [55]. As shown in Figure 7b–d, the levels of α-, γ-, and δ-tocopherol all decreased over time during the accelerated storage test, indicating continuous consumption of tocopherols under accelerated storage conditions. In the early stages of the accelerated storage test, tocopherol contents in some enzymatically degummed RO samples were slightly lower than those in crude oil, which may be attributed to losses occurring during the degumming process [56]. Additionally, ultrasonic cavitation may promote lipid oxidation [11], thereby accelerating tocopherol depletion. As the accelerated storage test progressed, the decline in tocopherol content became more pronounced in crude oil, whereas the degummed samples generally retained higher tocopherol levels, suggesting that the degumming treatments under the applied processing conditions were associated with reduced tocopherol loss. This may be attributed to the removal of phospholipids, metal ions, and other pro-oxidative impurities during the degumming process, which reduces the oxidation rate of the oil system and consequently slows tocopherol consumption. Among all treatments, the UEDAC group exhibited better retention of all three tocopherols during the accelerated storage test. Specifically, α-, γ-, and δ-tocopherol decreased from 169.86, 344.82, and 13.05 mg/kg to 137.06, 266.86, and 8.49 mg/kg, respectively. Overall, UEDAC-treated RO showed better tocopherol retention during accelerated storage, suggesting a greater preservation of endogenous tocopherols under these conditions.

#### 3.8.3. Phytosterols Analysis

Phytosterols are naturally occurring bioactive compounds in RO and possess important nutritional functions, such as regulating cholesterol metabolism [57]. As shown in Figure 7e, the phytosterol contents of all samples decreased during the accelerated storage test, indicating a gradual loss of phytosterols under accelerated storage conditions. At the initial stage of storage, phytosterol contents in the degummed oil samples were slightly lower than those in the crude oil. This trend is similar to that observed for β-carotene [58]. As the accelerated storage test progressed, the phytosterol content in crude RO decreased more markedly than in the treated samples. This may be because untreated crude oil contains higher levels of residual phospholipids, metal ions, and other gum impurities, which can promote lipid oxidation and thereby accelerate phytosterol degradation [50,56]. The UEDA and UEDC groups showed relatively higher phytosterol retention during storage, while the composite-treatment groups generally maintained higher phytosterol levels. The phytosterol content in the EDAC group was higher than that in the UEDA and UEDC groups but lower than that in the UEDAC group. Overall, these results suggest that UEDAC treatment was associated with better retention of phytosterols during accelerated storage.

## 4. Conclusions

This study established an ultrasound-assisted PLC/PLA_2_ composite enzymatic degumming process aimed at improving the degumming efficiency and quality stability of RO. Through single-factor experiments and response surface methodology optimization, the optimal processing conditions were determined as follows: moisture content of 4%, pH of 7.0, reaction temperature of 51 °C, ultrasonic output power of 70%, reaction time of 60 min, CaCl_2_ addition of 0.04%, and a PLC-to-PLA_2_ mass ratio of 3:1. Under these optimized conditions, the residual phosphorus content in RO was reduced to 4.55 ± 0.23 mg/kg, indicating good predictive reliability of the developed model. LC-MS/MS analysis revealed that the major phospholipid classes in crude RO were PC, PG, and PE, and the phospholipid removal rate reached 98.19% after UEDAC treatment, indicating effective phospholipid removal under the optimized conditions. Lipidomics analysis further indicated that UEDAC primarily promoted the degradation of GPs and regulated changes in GLs composition. Additionally, UEDAC effectively reduced residual calcium and magnesium contents in the oil while unsaturated fatty acids remained the predominant fatty acid fraction after degumming. During accelerated storage, UEDAC exhibited favorable quality stability, antioxidant capacity, and retention of β-carotene, tocopherols, and phytosterols. Future studies incorporating appropriate enzyme-free controls under identical processing conditions are expected to further elucidate the specific contribution of PLC/PLA_2_ and the synergistic effects of enzymatic hydrolysis and ultrasound-assisted processing. Overall, ultrasound-assisted PLC/PLA_2_ composite enzymatic degumming improved degumming efficiency and was associated with better preservation of oil quality during accelerated storage, providing theoretical support and a technical reference for the development of efficient enzymatic degumming processes for RO.

## Figures and Tables

**Table 1:** ANOVA of RSM experimental results.

Source	Sum of Squares	df	Mean Square	F-Value	*p*-Value	
Model	54.16	9	6.02	83.49	<0.0001	significant
A	2.30	1	2.30	31.92	0.0008	
B	3.65	1	3.65	50.57	0.0002	
C	0.5050	1	0.5050	7.01	0.0331	
AB	0.1296	1	0.1269	1.80	0.2218	
AC	4.14	1	4.14	57.46	0.0001	
BC	3.13	1	3.13	43.47	0.0003	
A^2^	10.30	1	10.30	142.85	<0.0001	
B^2^	18.77	1	18.77	260.39	<0.0001	
C^2^	7.21	1	7.21	100.06	<0.0001	
Residual	0.5045	7	0.0721			
Lack of Fit	0.2063	3	0.0688	0.9225	0.5066	not significant
Pure Error	0.2982	4	0.0746			
Cor.Total	54.67	16				

Mean = 6.99, C.V.% = 3.84, R^2^ = 0.9908, R^2^adj = 0.9789, R^2^pre = 0.9311, Adeq Precision = 25.4061.

**Table 2:** Effects of different processes on metal Ions.

Minerals (mg/kg)	Crude Oil	EDAC	UEDAC	UEDA	UEDC
Mg	53.68 ± 0.75 ^a^	6.46 ± 0.40 ^c^	3.28 ± 0.28 ^d^	2.57 ± 0.01 ^d^	7.32 ± 0.20 ^b^
Ca	166.15 ± 3.44 ^a^	41.40 ± 4.85 ^b^	22.06 ± 1.21 ^d^	24.56 ± 0.73 ^cd^	28.85 ± 1.08 ^c^

Results are expressed as the mean ± SD of three independent experiments (n = 3). Different lowercase letters within the same row indicate significant differences among treatments (*p* < 0.05).

**Table 3:** Effects of different processing methods on fatty acid composition and content.

Fatty Acid (% Total Fatty Acids)	Crude Oil	UEDAC	EDAC	UEDA	UEDC
C10:0	-	2.02% ± 0.002 ^c^	-	2.84% ± 0.001 ^b^	3.66% ± 0.003 ^a^
C14:0	0.42% ± 0.001 ^a^	0.31% ± 0.001 ^a^	0.33% ± 0.001 ^a^	0.34% ± 0.001 ^a^	0.41% ± 0.001 ^a^
C16:0	6.42% ± 0.009 ^ab^	5.70% ± 0.001 ^b^	6.23% ± 0.007 ^ab^	6.40% ± 0.004 ^ab^	7.09% ± 0.000 ^a^
C16:1	0.85% ± 0.002 ^ab^	0.76% ± 0.001 ^b^	0.87% ± 0.000 ^ab^	1.21% ± 0.003 ^a^	1.28% ± 0.003 ^a^
C17:1	0.25% ± 0.001 ^a^	0.21% ± 0.001 ^a^	0.18% ± 0.000 ^a^	0.18% ± 0.000 ^a^	0.24% ± 0.000 ^a^
C18:0	2.84% ± 0.003 ^b^	3.25% ± 0.005 ^b^	3.10% ± 0.001 ^b^	3.87% ± 0.003 ^a^	3.25% ± 0.001 ^b^
C18:1	55.19% ± 0.006 ^a^	53.97% ± 0.007 ^a^	54.37% ± 0.019 ^a^	50.92% ± 0.007 ^b^	53.81% ± 0.020 ^a^
C18:2	26.56% ± 0.012 ^a^	26.42% ± 0.012 ^a^	27.84% ± 0.015 ^a^	27.49% ± 0.010 ^a^	25.60% ± 0.016 ^a^
C18:3	4.32% ± 0.003 ^a^	4.46% ± 0.007 ^a^	4.08% ± 0.003 ^a^	3.94% ± 0.001 ^a^	3.12% ± 0.000 ^b^
C20:1	1.32% ± 0.002 ^a^	1.10% ± 0.001 ^ab^	1.03% ± 0.001 ^b^	1.10% ± 0.002 ^ab^	0.81% ± 0.001 ^b^
C22:1	0.59% ± 0.001 ^a^	0.48% ± 0.002 ^a^	0.56% ± 0.002 ^a^	0.42% ± 0.000 ^a^	0.52% ± 0.001 ^a^
C28:0	0.37% ± 0.001 ^b^	0.48% ± 0.001 ^ab^	0.54% ± 0.001 ^a^	0.41% ± 0.000 ^ab^	0.20% ± 0.000 ^c^
C30:0	0.85% ± 0.001 ^a^	0.85% ± 0.001 ^a^	0.87% ± 0.001 ^a^	0.88% ± 0.001 ^a^	-
SFA	10.91% ± 0.006 ^c^	12.60% ± 0.008 ^b^	11.07% ± 0.005 ^c^	14.73% ± 0.006 ^a^	14.61% ± 0.004 ^a^
MUFA	58.21% ± 0.009 ^a^	56.52% ± 0.003 ^a^	57.01% ± 0.017 ^a^	53.84% ± 0.013 ^b^	56.66% ± 0.018 ^a^
PUFA	30.88% ± 0.015 ^ab^	30.88% ± 0.005 ^ab^	31.92% ± 0.013 ^a^	31.43% ± 0.009 ^a^	28.72% ± 0.015 ^b^
UFA	89.09% ± 0.006 ^a^	87.40% ± 0.008 ^b^	88.93% ± 0.005 ^a^	85.27% ± 0.006 ^c^	85.39% ± 0.004 ^c^

Results are expressed as the mean ± SD of three independent experiments (n = 3). Different lowercase letters within the same row indicate significant differences among treatments (*p* < 0.05). SFA (saturated fatty acids, C10:0 + C14:0 + C16:0 + C18:0 + C28:0 + C30:0), MUFA (monounsaturated fatty acids, C16:1 + C17:1 + C18:1 + C20:1 + C22:1), PUFA (polyunsaturated fatty acids, C18:2 + C18:3), UFA (unsaturated fatty acid, MUFA + PUFA).

## Data Availability

The original contributions presented in this study are included in the article. Further inquiries can be directed to the corresponding author.
